# Risk Factors and Management of Prosthetic Joint Infections in Megaprostheses—A Review of the Literature

**DOI:** 10.3390/antibiotics13010025

**Published:** 2023-12-26

**Authors:** Marcos R. Gonzalez, Juan Pretell-Mazzini, Santiago A. Lozano-Calderon

**Affiliations:** 1 Division of Orthopaedic Oncology, Department of Orthopaedic Surgery, Massachusetts General Hospital, Harvard Medical School, Boston, MA 02114, USA; mgonzalez52@mgh.harvard.edu (M.R.G.); slozanocalderon@mgh.harvard.edu (S.A.L.-C.); 2 Miami Cancer Institute, Division of Orthopedic Oncology, Baptist Health System South Florida, Plantation, FL 33324, USA

**Keywords:** endoprosthesis, tumor prosthesis, PJI, one-stage, two-stage

## Abstract

Prosthetic joint infection (PJI) is the most common mode of failure of megaprostheses, yet the literature on the topic is scarce, and studies report conflicting data regarding the optimal treatment strategy. Patients with megaprostheses PJI are often immunosuppressed, and surgeons must balance the trade-off between treatment efficacy and morbidity associated with the surgery aiming for infection eradication. Our review on megaprostheses PJI focuses on two axes: (1) risk factors and preventative strategies; and (2) surgical strategies to manage this condition. Risk factors were classified as either unmodifiable or modifiable. Attempts to decrease the risk of PJI should target the latter group. Strategies to prevent PJI include the use of silver-coated implants, timely discontinuation of perioperative antibiotic prophylaxis, and adequate soft tissue coverage to diminish the amount of dead space. Regarding surgical treatment, main strategies include debridement, antibiotics, implant retention (DAIR), DAIR with modular component exchange, stem retention (DAIR plus), one-stage, and two-stage revision. Two-stage revision is the “gold standard” for PJI in conventional implants; however, its success hinges on adequate soft tissue coverage and willingness of patients to tolerate a spacer for a minimum of 6 weeks. DAIR plus and one-stage revisions may be appropriate for a select group of patients who cannot endure the morbidity of two surgeries. Moreover, whenever DAIR is considered, exchange of the modular components should be performed (DAIR plus). Due to the low volume of megaprostheses implanted, studies assessing PJI should be conducted in a multi-institutional fashion. This would allow for more meaningful comparison of groups, with sufficient statistical power. Level of evidence: IV.

## 1. Introduction

Advances in the medical management of bone and soft tissue tumors have favored limb-salvaging procedures over amputation [1,2]. Modular endoprostheses, or megaprostheses, have become the main reconstructive option after resection of primary bone tumors and skeletal metastases in cases where metastasectomy is indicated, such as oligometastatic bone diseases in renal, thyroid, and selected cases of breast carcinoma [3,4]. More recently, megaprostheses have also been used for non-oncologic indications, such as distal femoral fractures [5] and periprosthetic fractures [6] in the elderly and/or severely osteoporotic patients, as well as for revision surgeries when bone stock is not adequate [7,8]. Despite advances in materials and component design, implant failure remains higher than in conventional implants, with the majority of patients undergoing revision surgery.

In their 2011 landmark study, Henderson et al. described prosthetic joint infections (PJI) as the most common mode of megaprostheses failure [9]. Notably, studies have reported a 20–25% PJI rate in megaprosthetic reconstruction [10,11,12]. In comparison, the rate of PJI after total hip arthroplasty and total knee arthroplasty is 0.5–1% [13] and 0.5–2% [14,15], respectively. Besides the higher infection risk after implantation, megaprosthetic infection is harder to eradicate than infection occurring in conventional implants. Advancements in PJI treatment in the last decades have resulted in eradication rates above 90% for conventional implants treated with two-stage revision [16]. In contrast, similar improvements have not been reported in patients with megaprosthesis PJI, with eradication rates ranging from 50% [17] to 75% [18]. Notable differences in patient populations contribute to the lower eradication rate seen in megaprosthesis PJI. Patients undergoing elective joint arthroplasty typically exhibit fewer comorbidities, and there is a concerted effort to optimize their health before surgery, even if it involves delaying the procedure. Conversely, patients undergoing megaprosthesis reconstruction present a higher prevalence of comorbidities and are immunosuppressed. In these cases, delaying the surgery is not feasible due to the need to resume systemic therapy promptly. Additional challenges to the treatment of PJI in this population include extended operative time, and, in megaprostheses implanted for revision surgery, possible low-grade infections not detected when the revision is performed [19,20,21]. Moreover, due to the low volume of use of these implants, studies on megaprosthetic PJI are scarce and treatment strategies are not standardized [22,23]. This has led to conflicting evidence regarding the superiority of two-stage revision compared to one-stage revision or debridement, antibiotics, and implant retention (DAIR). In addition, in patients with a high comorbidity profile, surgeons often need to consider the trade-off between treatment efficacy and morbidity associated with the surgery aiming at infection eradication.

Our review aims to provide a concise, yet comprehensive, review of the literature on megaprostheses PJI. Therefore, we cover all important aspects of this condition, placing special emphasis on the prevention and treatment aspects.

## 2. Diagnosis of PJI

The diagnosis of PJI remains challenging because the definition is not standardized, as diagnostic criteria vary depending on the society establishing them [24,25,26,27]. Although all guidelines agree that ≥2 positive cultures for a microorganism or presence of a sinus tract communicating with the joint confirm the diagnosis of PJI, significant variability exists in cases that do not fulfill these criteria. Moreover, all diagnostic criteria were developed based on patients with PJI in conventional implants and were later extrapolated to megaprostheses. Since no studies have assessed the accuracy of these PJI definitions in megaprostheses PJI, their external validity remains unknown. We recommend, however, that future studies use the 2011 Musculoskeletal Infection Society criteria [27], the 2018 International Consensus Meeting (ICM) criteria [24], or the European Bone and Joint Infection Society [25] to diagnose PJI in megaprostheses.

When the PJI diagnosis is reached, it is important to define the chronicity of the infection. Adequately assessing whether the PJI is acute or chronic has profound implications on the microbiologic profile, mechanism, and infection, and effective surgical technique. While the exact cutoff for acute PJI remains controversial, ranging from 2 weeks to 90 days after index procedure, we consider that the two-week cutoff established by Zimmerli et al. is the most applicable to megaprosthetic PJI [28]. The larger metal surface area of megaprostheses likely accelerates the biofilm formation process, thus, requiring the use of a lower threshold in these implant types. At our institution, we classify megaprosthesis PJI as acute (less than 2 weeks), subacute (2 to 6 weeks), and chronic (more than 6 weeks). For PJI beyond the six-week mark (chronic), removal of the implant (modular components with or without stems) should be attempted. The decision to remove all components or leave the stems in situ is made on a case-by-case bias and depends on patient characteristics.

## 3. Risk of PJI in Megaprosthesis

Variability in PJI definition, perioperative antibiotic regimens, megaprostheses types, and patient comorbidity profiles across studies has resulted in a wide range of PJI rates following implantation, ranging from 0% [29,30] to 25% [12]. In a systematic review of 4838 patients undergoing reconstruction with a megaprosthesis after bone tumor resection, Racano et al. reported a weighted infection rate of 10% [31]. Notably, the authors underscored the significant heterogeneity among studies (I^2^ < 0.001). The broad timespan covered (1960s to 2010s), different prosthesis types used (silver-coated vs. uncoated titanium), and variations in antibiotic coverage and duration may partially explain these differences. Regarding antibiotics, the authors found that <50% of studies reported the antibiotic regimens administered, and only seven studies (15%) detailed the dose and drugs used [31]. Despite study differences, the risk of PJI in megaprostheses remains 5 to 10 times higher than that associated with conventional implants [13,14,15]. Surgeons should also factor in the anatomic segment the prosthesis replaces and its total metal surface area when assessing infection risk (Table 1). In Henderson et al.’s study, longer prosthesis, such as total humerus and total femur replacement, displayed the highest infection rates (18.8% and 11.5%, respectively) [9]. Although proximal tibia replacements also showed one of the higher PJI rates (15.1%), additional factors such as lack of soft tissue coverage need to be considered in this type of prosthesis. Notably, the majority of the risk factors mentioned in Table 1 also apply to patients treated with conventional implants. However, their prevalence is considerably lower in this population and pre-operative patient optimization, which often involves delaying the surgery to manage the underlying condition, is one of the cornerstones of total joint arthroplasty.

Although patients with megaprostheses are still at high risk of developing PJI, several improvements have been introduced in the last decades. These include use of bactericidal coating materials in megaprostheses [43], use of laminar air flow and aspiration suits intraoperatively to minimize airborne bacterial contamination [44], and enhanced soft tissue coverage techniques [39]. Finally, the prophylactic antibiotic regimens in tumor surgery (PARITY) demonstrated that the prolonged used of post-operative antibiotics beyond one day did not reduce the surgical site infection rate; rather, it increased the risk of antibiotic-related complications, with *C. difficile* colitis being the most prevalent (16%) [38].

## 4. Prevention of PJI

Treatment of megaprostheses PJI poses considerable challenges depending on the population that is being treated due to numerous factors, including poor soft tissue condition, immunocompromised state of patients, and the need to resume chemotherapy soon afterwards, among others. In addition to adequate surgical treatment selection, focus should be placed on strategies that seek to prevent PJI development after megaprostheses implantation. This requires a comprehensive assessment of risk factors for PJI, which can be stratified into modifiable and nonmodifiable risk factors (Table 1). The latter includes the use of chemotherapy, radiotherapy, immunocompromised state, comorbidity profile, soft tissue condition, and amount of resected tissue, while the former includes perioperative antibiotic regimen, soft tissue coverage strategy, operative time, and megaprosthesis alloy and coating selection, among others.

Regarding unmodifiable risk factors, use of systemic treatment, mostly chemotherapy, increases the risk of both developing a PJI and failing subsequent treatment [32]. Moreover, Donati et al. reported that patients on chemotherapy may only present PJI symptoms once treatment is finished [33,34]. Radiotherapy, which interferes with surgical wound healing, has also been recognized as a risk factor for megaprostheses PJI [18,36,37]. Radiotherapy also contributes to the poor state of the soft tissue, which is among the most important risk factors for developing a PJI as well as failure to treat them [41]. In addition, larger exposures and more extensive fascial resections have been described as predictors of PJI [39,42]. Notably, a recent secondary analysis of the PARITY dataset indicated that moderate to large fascial excision (≥1 cm^2^) was a risk factor for megaprostheses infection [42].

On modifiable risk factors, timely discontinuation of perioperative antibiotic prophylaxis is necessary to avoid antibiotic-related complications and prevent development of antibiotic resistance [45]. The results from the PARITY study, the first randomized controlled trial in orthopaedic oncology, show that prolonged postoperative intravenous antibiotic prophylaxis is not associated with lower rates of postoperative wound infections [38]. Surgeons and clinicians alike should, therefore, consider the uncertainty of benefits and the likely associated harm of prolonged antibiotic courses when deciding on antibiotic duration. Adequate soft tissue coverage is necessary to avoid dead spaces that increase the risk of PJI; this is particularly important in proximal tibia replacements, which have a higher failure rate than most megaprostheses [39,40]. In this realm, enhanced soft-tissue reconstruction techniques, such as gastrocnemius flap and free tissue transfers, have reduced the risk of infection [39,46,47]. We recommend that multidisciplinary approaches involving plastic surgeons be considered when complex soft tissue coverage is anticipated. Extended operative time has been associated with higher infection risk in most studies [20,21]. However, a recent study in oncologic megaprostheses found no difference in infection-free survival based on operative time [48]. Moreover, the authors reported that patients who underwent first revision for infection and did not develop a second failure had a longer median operative time. Although minimizing operative time in primary implantation should always be sought, Theil et al.’s findings suggest that, in revision surgery, a longer operating time, indicating a more thorough debridement, may be required. In addition, length of stay has been identified as a risk factor for PJI after megaprosthesis reconstruction [35]. In a secondary analysis of the PARITY study data, Slawaska-Eng et al. reported that each additional day of inpatient stay resulted in a 3% increase in the risk of PJI [35]. Although length of stay cannot be entirely modified, adequate post-operative protocols including physical therapy and optimization of anticoagulation and antibiotic regimens could potentially lead to shorter stays. Due to the extensive literature on megaprosthesis alloys and coatings, the following section focuses exclusively on this topic.

### 4.1. Megaprosthesis Alloys and Coatings

While antibiotics are highly effective at decreasing the number of bacteria, their efficacy diminishes once a biofilm has formed [49]. In such cases, successful treatment of the PJI requires removal of the implant, a procedure associated with substantial morbidity and additional bone loss [50]. Surface modification of implants is a strategy designed to prevent bacterial adhesion, colonization, and proliferation and biofilm formation. However, adequate selection of the antimicrobial coating is challenging, as the coating must inhibit bacterial activity without compromising the implant’s ability to integrate with bone tissue. Moreover, concerns about the development of antimicrobial resistance exist when pathogens are exposed to low minimum inhibitory concentrations for long periods.

#### 4.1.1. Implant Alloys

Most megaprostheses currently manufactured utilize one of two allows: cobalt–chrome (Co–Cr) and titanium [51]. Both in vitro and animal studies have demonstrated higher infection rates with the use of Co–Cr compared to titanium [52]. Likely explanations for these findings include the diminished respiratory burst of neutrophils when exposed to Co–Cr and the lower biocompatibility of Co–Cr alloys compared to titanium alloys [52]. Similar findings were seen in a clinical study on patients with megaprostheses, revealing an infection rate of 31.2% in the Co–Cr alloy group and 14.2% in the titanium alloy group (*p* < 0.01) [51]. Subsequent subgroup analysis highlighted that the difference in rates only occurred in chronic PJI, emphasizing the role of titanium suppressing biofilm formation. Consistent with these studies, the spine literature also reports a higher propensity for biofilm formation in Co–Cr alloy implants compared to titanium alloy implants [53].

#### 4.1.2. Antimicrobial Coatings

Implant coatings are usually classified into two types: (A) passive anti-adhesive coatings designed to prevent bacterial adhesion, and (B) active antimicrobial approaches aimed at killing bacteria [49].

While several materials, including polymeric compounds and biosurfactants, have been investigated for preventing PJI, silver stands out as the most utilized [54,55]. Silver, a heavy metal, has been favored due to its long-lasting antimicrobial properties against bacteria, fungi, and protozoa [54]. The antibacterial properties of this metal are attributed to the following mechanisms of action: (1) perforation of the cell wall and membrane; (2) inhibition of DNA transcription; (3) inactivation of bacterial proteins and disruption of ribosomal activity; (4) disruption of cellular respiration; and (5) leakage of nutrients and cellular components [49,56].

Currently, there are three silver-coated prostheses available on the market: MUTARS^®^ (Implantcast, Buxtehude, Germany), METS^®^ (Stanmore Implants Worldwide, London, UK), and Megasystem C^®^ (Waldemar Link GmbH & Co., Hamburg, Germany). In all three implants, none of the silver coating is applied on the articulating surface or on the prosthetic stems [57]. The efficacy of silver-coated megaprostheses preventing PJI was demonstrated in a recent systematic review and meta-analysis by Fiore et al. [58]. In a cohort of 755 patients, the authors reported PJI rates after implantation of 9.2% and 11.2% for silver-coated and uncoated megaprostheses, respectively [58]. Furthermore, a subgroup analysis focused on revision surgery related to PJI showed a reduction in infection cases in the silver-coated implant group. Among the studies included, Wafa et al. compared the microbiology of PJI in patients with silver-coated and uncoated megaprostheses [59]. Although no statistical analysis was performed, they reported that patients with uncoated megaprosthesis PJI had a higher rate of gram-negative PJI (37% vs. 30%) and polymicrobial PJI.

Although studies on heavy metal toxicity in this type of prosthesis are scarce, only minor local adverse effects have been reported [60]. Recent advancements in silver coating technology have shown the efficacy of on-demand release of silver ions using extracorporeal shockwaves, thereby reducing side effects and toxicity [61]. Surgeons should, therefore, consider using silver-coated implants in cases deemed at high risk of PJI or on instances where reimplantation follows PJI treatment.

## 5. Microbiology of PJI in Megaprosthesis

Despite the growing number of studies in megaprosthesis PJI, few of them describe the microorganism profile. In a systematic review, Racano et al. noted that only 27% (13/48) of studies evaluating PJI risk after megaprostheses implantation provided information on the associated microorganisms [31]. In those that did provide information, *Staphylococcus aureus* and coagulase-negative staphylococci (CoNS) were the most common organisms involved. In the largest megaprosthesis PJI cohort to date, Jeys et al. identified CoNS as the most common pathogen (48%) [36]. With about 80% of included PJI cases occurring in proximal tibia (35%) and distal femur replacement (42%), their results showcase the microbiology of knee PJI. Contrary to the prevailing literature, Sanders et al. found that multiflora and Gram-negative microorganisms predominated in PJI affecting pelvic endoprostheses after tumor resection [62]. They attributed these results to the different mechanism of bacterial contamination. Although the literature agrees that most acute PJIs occur due to intraoperative introduction of microorganisms, additional mechanisms such as contiguous spread via compromised soft tissue or hematogenous seeding have been described [28]. Discrepancies in PJI microbiology may also be explained by the timing of infection [63]. Acute PJIs predominantly involve high-virulence organisms like *Staphylococcus aureus* and Gram-negative bacteria, whereas chronic PJIs tend to be caused by low-virulence bacteria such as CoNs and *Cutibacterium* species. While neither study described the distribution of PJI (acute vs. chronic), Sanders et al. reported a significantly shorter time to PJI (1.2 months) [62] compared to Jeys et al.’s study (8.5 months) [36]. Therefore, the higher frequency of acute PJI in Sander et al.’s study may explain the higher rate of highly virulent organisms such as Gram-negative bacteria in their cohort. These findings suggest that microbiologic patterns in PJI may vary based on anatomic location of the implanted prosthesis and timing of the PJI.

While studies in conventional joint arthroplasty have highlighted worse outcomes in Gram-negative and fungal PJI [64,65], the impact of microorganism profile on treatment success rates of megaprostheses PJI remains unknown. Furthermore, the few studies available on the subject are characterized by a heterogeneous population and the lack of controlling for potential confounders [36,66,67]. Morii et al. examined the impact of specific bacteria on clinical symptoms and inflammatory parameters in 57 patients with megaprostheses PJI around the knee (distal femur and proximal tibia replacements) [66]. Their analysis revealed that *Staphylococcus aureus* and methicillin-resistant *Staphylococcus aureus* (MRSA) were associated with discharge and pus and elevated CRP values, while *Staphylococcus epidermidis* was associated with mild inflammatory parameters. Ercolano et al. also assessed treatment success by microorganism and reported a failure rate of 60% (3/5) for MRSA, 62.5% (5/8) for polymicrobial infections, 46% (6/13) for single-organism infections, 50% (1/2) for fungal organisms, and 33% (1/3) for culture-negative PJI [66]. However, their findings were based on simple proportions, and no statistical analysis comparing groups was performed. The association between polymicrobial PJI and worse outcomes remains controversial, with Jeys et al. reporting similar treatment success rates between this cohort and single-organism PJI (HR = 1.1, *p* = 0.6) [36].

Although a number of studies report on the microbiology of megaprosthesis PJI, the robustness of their findings is limited by (1) significant cohort heterogeneity, and (2) lack of a standard definition for PJI treatment success. The literature suggests that patients with PJI caused by MRSA and fungal pathogens may potentially have worse outcomes. However, caution is warranted in interpreting these findings due to the retrospective nature and low sample size of the available studies.

## 6. Treatment of PJI

The limited literature on the surgical management of megaprostheses PJI has resulted in a lack of adequate standardization of treatment strategies. Indeed, several studies label surgeries in which the stems are retained as one-stage revisions [22,23]. Moreover, a clear definition of PJI recurrence or treatment failure is absent, as most studies report treatment success rates regardless of whether patients receive chronic antibiotic suppression. The prevalence of comorbidities and a history of immunosuppression further restrict surgical options, requiring surgeons to carefully balance treatment efficacy and associated morbidity.

### 6.1. Debridement, Antibiotics, and Implant Retention (DAIR)

DAIR procedures are strictly used in early PJI and involve debridement of all necrotic tissue with or without exchange of the modular components (i.e., liners and heads) [68]. In the conventional arthroplasty literature, DAIR has a reported PJI treatment success rate of 60% [69]. In megaprostheses PJI, DAIR is performed in patients with acute PJI who may not tolerate the morbidity associated with revision surgery. Studies in this population have reported a treatment success rate ranging from 40% to 75% (Table 2) [17,67,70,71,72]. Variations in PJI microbiology, antimicrobial resistance patterns, and megaprostheses type may explain the differences in success rates between studies. Ercolano et al.’s study, which reported a 40% treatment success rate with DAIR, had a high prevalence of more aggressive pathogens, such as MRSA (16%), fungal pathogens (6%), and polymicrobial infections (26%) [67]. Identification of the causative organism of the PJI before choosing the surgical strategy is critical, as previous research has reported that for certain MDR or XDR Gram-negative organisms, DAIR is associated with higher failure rates compared to two-stage revision, regardless of surgical timing [73]. Similar findings have been reported in fungal PJI, with DAIR being a risk factor for PJI recurrence compared to two-stage revision [64].

When DAIR is considered, surgeons should weigh the option of modular component exchange (DAIR plus). However, only one study in megaprostheses PJI compared treatment success rates in patients treated with DAIR and those treated with DAIR plus [72]. The authors reported a higher treatment success rate for DAIR plus (68%) compared to standard DAIR (50%); however, this difference was not significant due to the lower number of patients (*n* = 33) [72]. Additionally, subgroup analysis revealed that treatment success with DAIR was highest in acute postoperative PJI (82%); for acute hematogenous and chronic PJI, success rates were 44% and 57%, respectively. Another study by Holzer et al. reported a 78% success rate eradicating a megaprostheses PJI with DAIR plus [23]. This study, however, displayed several limitations as the authors did not include a standard DAIR control group and incorrectly labeled their surgery as one-stage revision. Since the stems were not revised intraoperatively, their surgical strategy constitutes, by definition, a DAIR plus.

We consider that in patients with acute PJI with drug-sensitive pathogens, DAIR can be considered as definite treatment. When performed, the modular components should be exchanged, as DAIR plus is associated with a higher treatment success rate (Table 2). Surgeons should, however, consider that up to 50% of patients may develop recurrence and additional debridement procedures may be required.

### 6.2. One-Stage and Two-Stage Revision

Removal of the prosthesis is required in chronic infections that develop mature biofilm and in infections caused by highly resistant microorganisms [68]. In the conventional arthroplasty literature, both one-stage and two-stage revisions are well-described treatments with success rates above 80% [75,76]. In megaprostheses PJI, however, results treatment success rates range from 45% to 47% for one-stage revisions [36,67], and from 62% to 75% in two-stage revisions (Table 2) [22,36,67,71]. Caution should be exerted when interpreting these results, since multiple studies labeled their surgeries as one-stage revision even though only the modular components were exchanged [22,23,74]. However, these constitute DAIR plus procedures and are displayed as such in Table 2.

One-stage revision procedures offer several benefits, including the avoidance of subjecting patients to an extended period with a cement spacer and decreased mobility and quality of life, reduced morbidity due to the need of single operation, and shorter inpatient stays resulting in diminished costs [74]. The first point is critical, as patients often do not tolerate having an antibiotic for at least 6 weeks and request earlier reimplantation. In our experience, the recommended 6 weeks between spacer insertion and reimplantation is often not feasible in megaprostheses PJI, with studies in this population reporting a mean time of 10 weeks between stages [18,37]. While the literature reports comparable treatment success rates between one-stage revision and DAIR procedures, this comparison is affected by selection bias. Patients with chronic PJI, which are harder to eradicate, are more likely to be treated with one-stage revision than DAIR. In our opinion, one-stage revision may only be considered when the microorganism is known, and patients have good soft tissue coverage. When performed, one-stage revision must include thorough debridement and exchange of all components, including well-fixed stems. The limited literature on one-stage revisions likely reflects the reasoning that, when surgeons decide to perform the highly morbid stem removal process, they would rather perform a two-staged procedure than reimplant a new megaprostheses in the same surgery.

**Figure 1:**
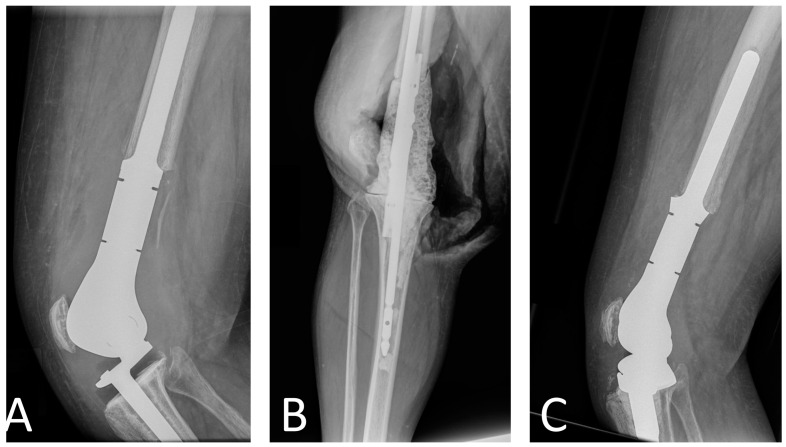
Radiographs of patient with distal femur replacement PJI treated with two-stage revision. (**A**) Pre-explantation radiographs, (**B**) intraoperative radiographs of the spacer with two tibial nails, and (**C**) post-reimplantation radiographs.

Two-stage revision is the gold standard for the management of megaprostheses PJI. This surgical strategy requires removal of all components, thorough soft tissue debridement, and placement of a cement spacer. Due to the morbidity of removing fixed components, some surgeons have advocated for a partial two-stage revision with stem retention, citing positive outcomes [37]. However, these findings stem from a single study that included 11 patients treated with this procedure and 4 patients with complete two-stage revision. In our opinion, retention of the stems does not allow for complete PJI eradication, and more recent data underscore this point [22]. Sigmund et al. analyzed the outcomes of 32 patients treated with two-stage revision for megaprostheses PJI and reported a 77% success rate for patients treated with complete stem removal and 36% for those with incomplete stem removal [22]. Regarding spacer construction for PJI involving megaprostheses around the knee, we recommend the use of two tibial nails, which serve as a scaffold for the antibiotic-loaded cement (Figure 1). Alternative spacer manufacturing techniques using Kuntscher nails and cement guns have been described [18]. Unless contraindicated, combination of 3 g of vancomycin and 3.6 g of tobramycin per 40 g package of bone cement is recommended [72]. Given the large size of these defects and the impossibility to use all cement bags with antibiotics, we recommend that the antibiotic cement is used at the bone–spacer interface and, secondarily, in the periphery of the spacer. A key feature in two-stage revisions is to ensure that a spacer of sufficient size is used to preserve the cavity in which the new prosthesis will be reimplanted in the second stage [18]. As patients with megaprostheses PJI often require higher doses of local antibiotics than those used in conventional PJI, surgeons should also be aware of the high risk of post-operative acute kidney injury (AKI) [77]. Underlying chronic kidney disease, high baseline creatinine, low preoperative hemoglobin, and blood transfusion requirement have been identified as risk factors for AKI after spacer insertion [78]. Although no specific time to reimplantation has been established, Jeys et al. recommended leaving the spacer in place for a minimum of six weeks [36]. Moreover, they recommended performing reimplantation only if no organism grew after three weeks of culture of periprosthetic aspirate. An antibiotic-free interval before joint aspiration (antibiotic holiday) of 2 weeks should be considered to minimize the number of false negative results [79].

**Figure 2:**
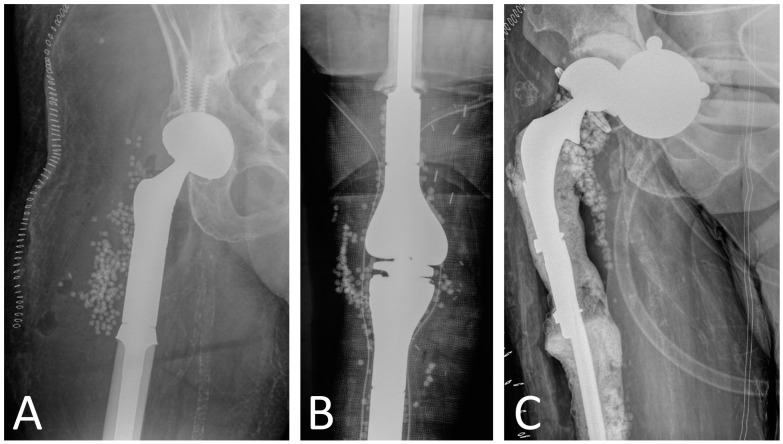
Use of calcium sulfate beads in megaprosthesis PJI as adjunct to surgical treatment in (**A**) proximal femur replacement, (**B**) distal femur replacement and proximal tibia replacement, and (**C**) proximal femur replacement. Patients in (**A**,**B**) underwent one-stage revision, while patient in Figure 2C had a two-stage revision.

Although spacers seek to locally deliver antibiotics at high doses, studies have reported a rapid reduction to subtherapeutic levels within 24 h of implantation [80]. Absorbable calcium sulfate (CS) beads have been promoted as an alternative to traditional spacers for local antibiotic delivery. Studies have reported that CS beads provide superior elution characteristics and higher sustained antibiotic concentrations than polymethyl methacrylate (PMMA) [81,82]. Despite their superior profile in in vitro studies, clinical studies examining the use of CS beads in conventional implants for acute or acute hematogenous PJI have shown no improvement in infection eradication rates when added to DAIR procedures [83,84]. In megaprostheses PJI, there is a single study by Donati et al. in the 1990s comparing gentamicin–PMMA beads and antibiotic-impregnated cement spacers in terms of PJI treatment success [33]. This study, however, was published in the 1990s, prior to many of the surgical advances in megaprostheses and did not analyze the use of CS beads, only PMMA beads. Moreover, gentamicin–PMMA beads cannot carry other antibiotics and are, therefore, not effective against aminoglycoside-resistant bacteria. Considering the lack of studies investigating CS beads in megaprostheses (Figure 2) and the lack of information on their complication profile, we consider that their routine use is not justified.

### 6.3. Amputation

PJI in total knee arthroplasty may result in above-knee amputation in 4% to 8.5% of cases [85,86]. In the megaprostheses PJI, this rate has been reported to be as high as 20% for bacterial PJI and 50% for fungal PJI [72,87]. Amputation, however, may be performed as both a primary PJI treatment or a last resort surgery after multiple failed procedures. Jeys et al. reported that 32% of their cohort was initially treated with amputation upon PJI diagnosis and 98% of them eradicated the infection [36]. Additional studies have also reported a 100% success rate when amputation is performed as initial surgery (Table 2) [36,71]. Although functional outcomes are significantly better with limb-sparing procedures [88], high amputation rates underscore how critical it is to achieve rapid infection control in this population. Most patients undergo megaprostheses reconstruction after resection of a bone tumor and need to restart adjuvant chemotherapy rapidly after surgery. Since discontinuation of antineoplastic drugs upon PJI onset may affect patient survival, surgical treatment seeks to achieve rapid and effective infection control so systemic treatment can be resumed. Therefore, a lower threshold for amputation as either primary surgery or treatment of PJI recurrence is considered.

While assessing the treatment efficacy and morbidity of each procedure is critical, surgeons should also consider the long-term functional outcomes and expected quality of life. Prior studies on conventional implants have reported lower patient-reported outcomes after PJI treatment [89], similar improvement in quality of life between patients treated with DAIR for a PJI and those without infection [90], and lower quality of life scores in two-stage revision for PJI compared to one-stage revision [91]. However, in megaprosthesis PJI, no studies have assessed how various surgical procedures influence long-term outcomes and quality of life. Therefore, future studies in megaprostheses patients should prioritize evaluating the impact of PJI on well-being and the performance of different procedures in this context.

## 7. Conclusions

Risk of PJI in megaprostheses is significantly higher than in conventional implants, and treatment is associated with high rates of treatment failure. Optimizing PJI prevention and treatment strategies is paramount to maximize the chances of successful treatment. Limitations of current studies include the lack of standardized PJI diagnosis criteria and inconsistent definition of treatment strategies. We recommend that future studies use the 2011 Musculoskeletal Infection Society criteria or the 2018 International Consensus Meeting criteria to diagnose PJI. For treatment, to define a surgery as one- or two-stage revision, removal of all prosthesis components must occur.

Our review focused on the risk of PJI after megaprostheses implantation and the available surgical strategies for those. We emphasized the importance of identifying risk factors for PJI and addressing those that are modifiable. Whenever DAIR is considered, exchange of the modular components should be performed (DAIR plus). In our experience, patients with acute PJI and well-fixed stems should be treated with DAIR plus. Two-stage revision is the gold standard and should be the first-line option for patients with chronic megaprostheses PJI and loose stems.

Due to the low volume of megaprostheses implanted, studies assessing PJI should be conducted in a multi-institutional fashion. This would allow for more meaningful comparison of groups, with sufficient statistical power.

## Figures and Tables

**Table 1:** Risk factors for megaprosthesis prosthetic joint infection.

Unmodifiable		Modifiable	
Risk Factor(s)	Reference(s)	Risk Factor(s)	Reference(s)
Chemotherapy	[32,33,34]	Length of stay	[35]
Radiation therapy	[18,36,37]	Perioperative antibiotic regimen	[38]
Immunosuppression	[32,34]	Adequate soft tissue coverage (gastrocnemius flap, free flaps)	[39,40]
Soft tissue condition	[23,41]	Deep Hemovac drain ^α^	[42]
Tibial site	[9,36,39]	Operative time	[20,21]
Amount of fascia excised	[42]		

^α^ Although a protective factor in separate multivariate logistic regression, it was not a significant factor in final multivariate logistic regression (*p* = 0.06).

**Table 2:** Studies reporting the treatment success of different treatment modalities.

**Author**	**Treatment** **Strategy**	**Population**	**Sample (*n*)**	**Study Sample (*n*)**	**Types of Megaprosthesis**	**Success Rate**
Ercolano et al. [67]	DAIR	Oncologic	15	31	DFR (39%), PFR (35%), PTR (10%), TFR (10%), THR (6%) ^α^	40%
Allison et al. [71]	DAIR	Oncologic	-	43	DFR (56%), PFR (23%), DFR (21%) ^α^	42%
Lee et al. [70]	DAIR	Oncologic	11	18	PTR (45%), DFR (36%), PFR (9%), pelvis (9%)	45%
Sukhonthamarn et al. [72]	DAIR	Non-oncologic	8	33	DFR (55%), PFR (45%)	50%
Peel et al. [17]	DAIR	Oncologic	9	17	DFR (75%), saddle prosthesis (25%)	75%
Sigmund et al. [22]	DAIR plus	Oncologic	61	81	DFR (51%), PTR (31%), PFR (15%), other (3%) ^α^	51%
Funovics et al. [74]	DAIR plus	Oncologic	8	12	PFR (100%)	63%
Sukhonthamarn et al. [72]	DAIR plus	Non-oncologic	19	33	DFR (55%), PFR (45%)	68%
Allison et al. [71]	DAIR plus	Oncologic	-	43	DFR (56%), PFR (23%), DFR (21%) ^α^	70%
Holzer et al. [23]	DAIR plus ^β^	Oncologic	18	18	DFR (44%), PTR (22%), PFR (17%), THR (11%), TFR (6%)	78%
Ercolano et al. [67]	One-stage	Oncologic	11	31	DFR (39%), PFR (35%), PTR (10%), TFR (10%), THR (6%) ^α^	45%
Jeys et al. [36]	One-stage	Oncologic	33 ^δ^	136	PTR (42%), DFR (35%), PTR (13%), pelvis (8%), PHR (2%) ^α^	47%
Peel et al. [17]	Two-stage	Oncologic	4	17	DFR (100%)	50%
Allison et al. [71]	Two-stage	Oncologic	-	43	DFR (56%), PFR (23%), DFR (21%) ^α^	62%
Sigmund et al. [22]	Two-stage ^γ^	Oncologic	16	81	DFR (51%), PTR (31%), PFR (15%), other (3%) ^α^	62%
Jeys et al. [36]	Two-stage	Oncologic	58 ^δ^	136	PTR (42%), DFR (35%), PTR (13%), pelvis (8%), PHR (2%) ^α^	72%
Flint et al. [37]	Two-stage ^ε^	Oncologic	11	15	DFR (60%), PTR (27%), PFR 13%) ^α^	73%
Ercolano et al. [67]	Two-stage	Oncologic	4	31	DFR (39%), PFR (35%), PTR (10%), TFR (10%), THR (6%) ^α^	75%
Grimer et al. [18]	Two-stage	Oncologic	34	34	DFR (50%), PTR (29%), PFR (15%), other (6%)	75%
Jeys et al. [36]	Amputation	Oncologic	43 ^δ^	136	PTR (42%), DFR (35%), PTR (13%), pelvis (8%), PHR (2%) ^α^	98%
Sigmund et al. [22]	Amputation	Oncologic	4	81	DFR (51%), PTR (31%), PFR (15%), other (3%) ^α^	100%
Allison et al. [71]	Amputation	Oncologic	-	45	DFR (56%), PFR (23%), DFR (21%) ^α^	100%

^α^ Reflects data from the entire cohort study, not specific to this treatment strategy; ^β^ described by authors as one-stage, classified as DAIR plus since the stems were not revised; ^γ^ authors described that 44% (14/32) of the two-stage revisions had incomplete removal (at least one well fixed stem); ^δ^ authors assessed treatment success rate by surgery rather than by patient (restricted to the first treatment of the PJI). This explains the higher number of procedures compared to number of patients with PJI (*n* = 136); ^ε^ authors described that in 67% (10/15) of the two-stage revisions, the stems were left in situ. DFR: distal femur replacement; PFR: proximal femur replacement; PHR: proximal humerus replacement; PTR: proximal tibia replacement; DAIR: debridement, antibiotics, and implant retention; TFR: total femur replacement; THR: total humerus replacement.
